# Mitigating spread of contamination in meat supply chain management using deep learning

**DOI:** 10.1038/s41598-022-08993-5

**Published:** 2022-03-23

**Authors:** Mohammad Amin Amani, Samuel Asumadu Sarkodie

**Affiliations:** 1 School of Industrial Engineering, College of Engineering, University of Tehran, Tehran, Iran; 2 Nord University Business School (HHN), Post Box 1490, 8049 Bodø, Norway

**Keywords:** Engineering, Computer science, Scientific data, Statistics

## Abstract

Industry 4.0 recommends a paradigm shift from traditional manufacturing to automated industrial practices, especially in different parts of supply chain management. Besides, the Sustainable Development Goal (SDG) 12 underscores the urgency of ensuring a sustainable supply chain with novel technologies including Artificial Intelligence to decrease food loss, which has the potential of mitigating food waste. These new technologies can increase productivity, especially in perishable products of the supply chain by reducing expenses, increasing the accuracy of operations, accelerating processes, and decreasing the carbon footprint of food. Artificial intelligence techniques such as deep learning can be utilized in various sections of meat supply chain management––where highly perishable products like spoiled meat need to be separated from wholesome ones to prevent cross-contamination with food-borne pathogens. Therefore, to automate this process and prevent meat spoilage and/or improve meat shelf life which is crucial to consumer meat preferences and sustainable consumption, a classification model was trained by the DCNN and PSO algorithms with 100% accuracy, which discerns wholesome meat from spoiled ones.

## Introduction

Supply chain management (SCM) remains one of the most critical factors in the development of various industries. Perishable product supply chain management has attracted scientific attention in the last two decades due to the rising demands for perishable products. It is estimated that 1.3 billion tons (i.e., economic value of over $1 trillion) of produced food is wasted annually, of which 25% of this wasted food can feed the 795 million malnourished people in the world^1^. Meat is a highly perishable product that accounts for 13% of food waste^2^. Besides, meat spoilage contributes to one-third of emissions (i.e., the greenhouse gas emissions like CO2) and 75% of land area used by wasted food^3^. Although traditional techniques which are prone to human errors, such as manually monitoring and controlling or utilizing non-intelligent systems, are still employed in meat supply chain management across countries, especially in developing economies^4^. However, the industry 4.0 paradigm recommends the automation of processes using novel technologies^5^ and new technologies such as artificial intelligence to enhance sustainable performance. Artificial intelligence can be employed to increase productivity while decreasing expenses and improving responsible production and consumption, as expounded in the Sustainable Development Goal (SDG) 12^6,7^.

The SDG12 indicates efficient utilization of resources, developing energy productivity, sustainable infrastructure, green works, and guaranteeing a good life^8^. The SDG12 outlines multiple targets, including sustainable management and food waste reduction during production and supply chain^9^. Yet, the risk is unavoidable in perishable product supply chain management. The rising chemical and biological risk of contamination associated with highly perishable meat products increases meat spoilage, hence, affecting the sustainability of perishable products in the supply chain^10^. Meat has a short life, and its value decreases over time due to exposure to rot and damage during logistics, transport, and storage––which indicates the susceptibility, complexity, and uncertainty of meat and other perishable products in the supply chain^11,12^.

Several factors affect the perishability dynamics of meat, namely pre-slaughter operations, the welfare of animals on the farm (including quality of food given to livestock), during transportation to a slaughterhouse, age of livestock in the slaughtering process^13^, and good hygienic practice (GHP) in the slaughtering process can avoid bacteria infection^14^. Moreover, other drivers that are effective in the perishability and shelf life of meat products include proper packaging (i.e., vacuum or modified atmosphere packaging (MAP)), handling techniques during slaughtering, optimal temperature control during transportation, retail, and consumer sections, distribution conditions (i.e., time, temperature, and type of vehicles), preservation techniques, and storage^15–17^. The proximity of spoiled meat to wholesome meat is one critical factor of meat spoilage––where bacteria (i.e., *Brochothrix thermosphacta*, *Carnobacterium spp.*, *Enterobacteriaceae, Lactococcus lactis, Lactobacillus spp., Leuconostoc spp., Pseudomonas spp, and Shewanella putrefaciens*) can grow and spread to the meat by extrinsic factors, processes (transporting and packaging) and environmental conditions (such as changing humidity and temperature)^18,19^. Therefore, controlling and monitoring meat production during the various stages of the supply chain to manage this risk is crucial to achieving food safety^20^. Thus, new intelligent technologies such as smart containers, artificial intelligence, or IoT methods are required to mitigate post-harvest losses, decrease lead times, and control the perishability dynamics in meat supply chain management^12^.

Industry 4.0 proposes a paradigm shift from traditional manufacturing to automated industrial operations by utilizing novel technologies, including artificial intelligence^21^. The provision of food to meet demand due to the ever-increasing population has become a global issue^22^. However, traditional agricultural supply chains may fail to meet global demand unless they are optimized by embedding intelligent technologies in the production function^6^. Enterprises can address clients' new demands and supply challenges while maintaining expectations in efficiency such as inter alia, online-enabled transparency, easy access to a multitude of options, and constant changes in stock-keeping unit (SKU) portfolio. The efficiency of a supply chain is amplified by automating both physical tasks and planning^23^. Machine learning is a method widely implemented to find patterns and linear and non-linear relationships between different variables, and it has various subcategories such as Classification, Regression, or Clustering^24–26^, which can be employed to analyze and help make decisions^27^. Machine learning and deep learning, which are subcategories of artificial intelligence, facilitate the generation of actionable intelligence by processing gathered data to improve manufacturing productivity without substantially altering recommended resources^28^.

The use of artificial intelligence in the management of perishable products in the supply chain has received much attention. Shahbazi and Byun^29^ employed novel technologies, including blockchain, machine learning, and fuzzy logic to develop a better traceability system in the supply chain that addresses several factors of the perishable food supply chain, including evaporation, weight, warehouse transactions, or shipping time, which enhances the shelf life of perishable foods. Alfian, Syafrudin^30^ proposed the radio frequency identification (RFID) technology for traceability of perishable foods, machine learning to detect the direction of passive RFID tags, and IoT to control temperature and humidity during storage and transportation, which this integration between these technologies enhance the efficiency of the traceability system. Barbon, Barbin^31^ used machine learning algorithms to predict the quality of chicken based on near-infrared (NIR) spectra data by analyzing chicken.

Deep learning techniques such as deep convolutional neural networks are utilized to automate food manufacturing and supply chain management tasks based on the industry 4.0 paradigm. Al-Sarayreh^32^ proposed an integrated system of hyperspectral imaging and deep learning techniques to assess the quality of various food products such as meat. Zhang^33^ presented a convolutional neural networks (CNN) model for vibrational spectral analysis, which measures specific chemical bonds of atoms and molecules. The estimated model achieved 99.01% accuracy (better performance than other theoretical techniques) on a meat dataset that consists of chicken, pork, and turkey. Al-Sarayreh^34^ employed the CNN algorithm to build a model for detecting lamb, beef, and pork meat adulteration (i.e., adding another type of meat that has a lower price to the meat pack). The model classified meats with 94.4% accuracy. Liu^35^ utilized a CNN model to detect and analyze the complex matrices of various foods such as meat products, aquatic products, cereal products, fruits, and vegetables.

In contrast, existing literature that detects and mitigates the potential spread of contamination among perishable foods is limited. Here, due to the effect of bacteria-driven meat spoilage in spreading from rotten meat into healthy meats and leading to contamination, this study employs artificial intelligence based on industry 4.0 and SDG12 paradigms to automate the controlling and monitoring process of meat production by detecting spoiled meats at various stages of the meat supply chain management. This is useful for improving the meat shelf life, reducing harm and health-related risk to consumers, and mitigating food and economic waste. Therefore, a deep convolutional neural network is employed as a classifier to increase productivity and reduce costs, whereas the PSO algorithm tunes the classifier hyperparameters.

The remaining sections of this paper are organized as follows—"Methodology " proposes a deep convolutional neural network model and a PSO algorithm utilized for hyperparameter tuning. The experimental results are presented in "Data acquisition", whereas the discussion and conclusion are presented in "Particle swarm optimization algorithm" and "Deep convolutional neural networks", respectively.

## Methodology

This section describes a popular optimization algorithm, which is employed to adjust the hyperparameters in the deep learning model. Moreover, the deep convolutional neural network and its layers are presented herein.

### Data acquisition

The dataset utilized in this study was adopted from previous research^36^. This dataset contains two classes, wholesome and spoiled red meat samples collected from a supermarket in Turkey. The dataset has 1896 images in total, with 948 per class gathered via an IP camera with an image resolution of 1280 × 720.

### Particle swarm optimization algorithm

Particle Swarm Optimization (PSO) is a population-based stochastic optimization algorithm that is inspired by the bird's swarm social behavior. This algorithm is a member of the Swarm Intelligence (SI) that arises from research based on creatures living as a group. This group members have little or no insight but can operate complicated works by interacting with each other^37^.

In this algorithm, a population of random particles is first determined, and each of the individuals is assigned a velocity and position. The historical behavior of each particle and its neighbors, while they move through the search area, adjusts the paces. The new position is updated by the accelerations of the next step and the current situation. Therefore, the particles move towards the suitable search space along the searching process and consequently closer to the optimum spot. Each particle that has its position, acceleration, and fitness value displays a solution in search space. Every particle goes to its best place and best position of particle swarm at each iteration. The movement of particles depends on the speed change calculated using Eq. (1), expressed as:

1 $${\overrightarrow{V}}_{ij}\left(t+1\right)= {\varphi }_{1}{r}_{1}\left({\overrightarrow{p}}_{ij}\left(t\right)- {\overrightarrow{x}}_{ij}\left(t\right)\right)+{\varphi }_{2}{r}_{2}\left({\overrightarrow{p}}_{gi}\left(t\right)- {\overrightarrow{x}}_{ij}\left(t\right)\right)+W {\overrightarrow{V}}_{ij}\left(t\right)$$

where ${\overrightarrow{V}}_{ij}\left(t+1\right)$ is particle *i* speed on the *jth* dimension at *t* + *1* iteration, ${\overrightarrow{V}}_{ij}\left(t\right)$ is particle *i* speed on the *jth* dimension at *t* iteration, ${\overrightarrow{x}}_{ij}\left(t\right)$ is particle *i* position on the *jth* dimension at *t* iteration, ${r}_{1}$ and ${r}_{2}$ are random numbers between (0,1), *W* is a constant, ${\varphi }_{1}$ and ${\varphi }_{2}$ are constants that are known as velocity coefficients, ${\overrightarrow{p}}_{ij}\left(t\right)$ is the best position of a particle, and ${\overrightarrow{p}}_{gi}\left(t\right)$ is the best position of the particle swarm. The new position of a particle is calculated using Eq. (2).

2 $${\overrightarrow{x}}_{ij}\left(t+1\right)= {\overrightarrow{x}}_{ij}\left(t\right)+ {\overrightarrow{V}}_{ij}\left(t+1\right)$$

**Figure 1:**
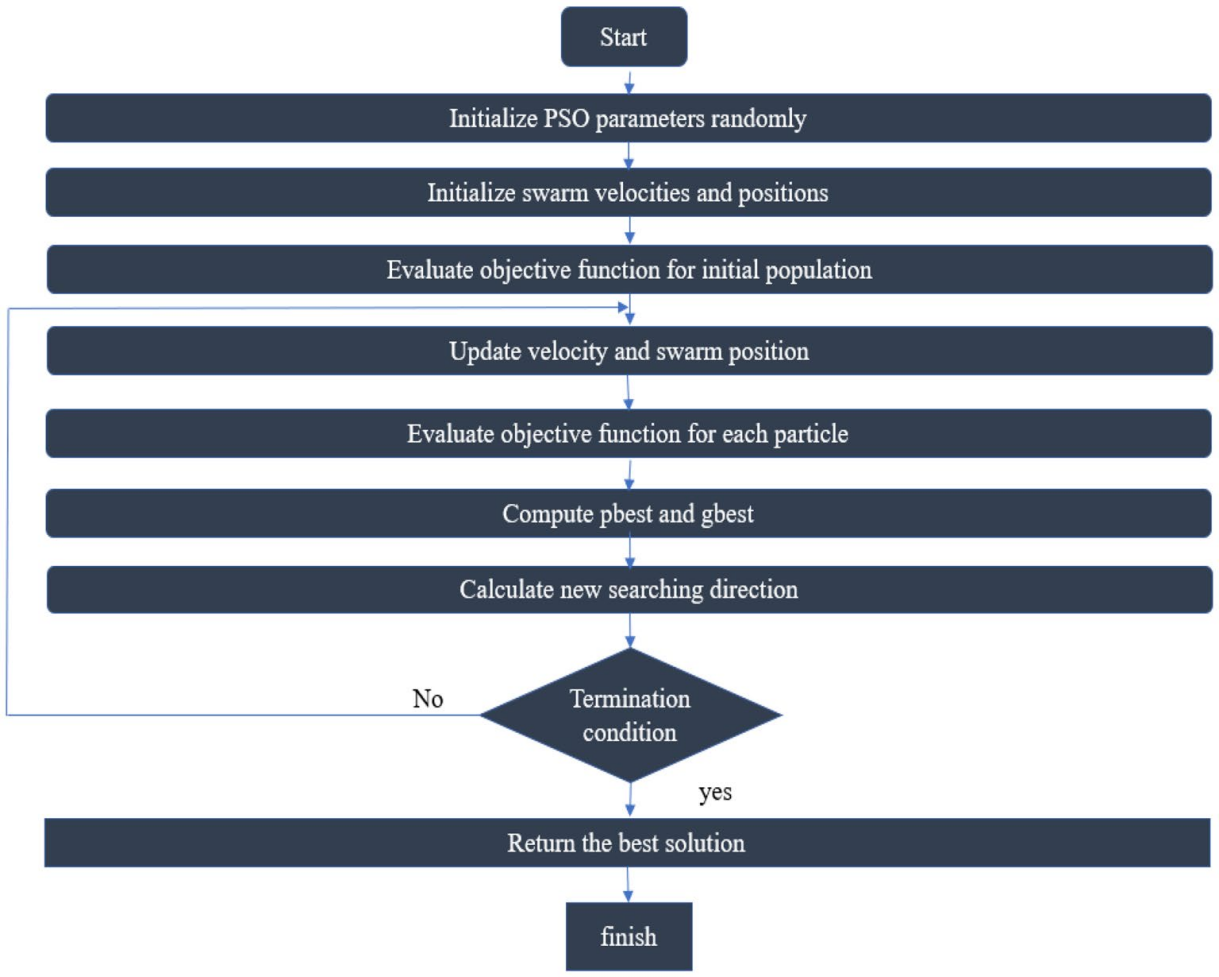
The PSO optimization algorithm flowchart. Source: Authors' construction using Powerpoint.

Figure 1 illustrates the PSO optimization algorithm flowchart. This paper employs the PSO algorithm to tune the hyperparameters in the deep convolutional neural network model.

### Deep convolutional neural networks

**Figure 2:**
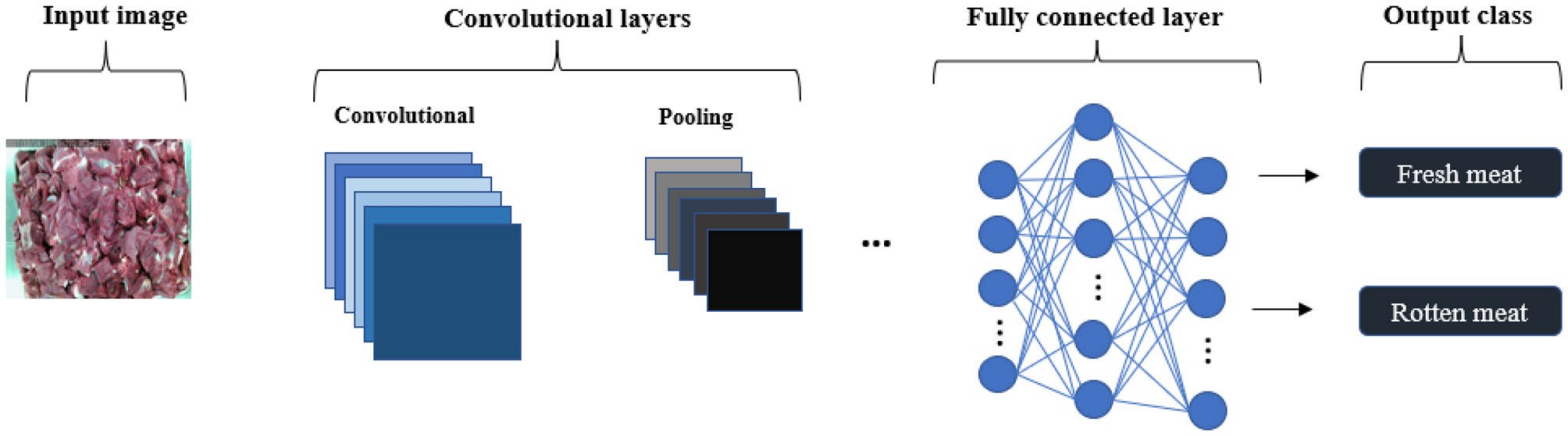
The DCNN image classification process. Source: Authors' construction using Powerpoint.

Deep learning is a subcategory of machine learning algorithms with various architectures, such as, *inter alia*, deep neural networks (DNNs), CNNs, and recurrent neural networks (RNNs). CNN is one of the common algorithms utilized for image classification and recognition. Input, hidden, and fully connected layers are elements of a CNN's construction. Figure 2 illustrates the deep convolutional neural networks (DCNN) image classification process utilized herein. The hidden layers comprise convolutional and pooling layers. In this algorithm, the pixel values of the image are turned to an array as input for CNN.

**Figure 3:**
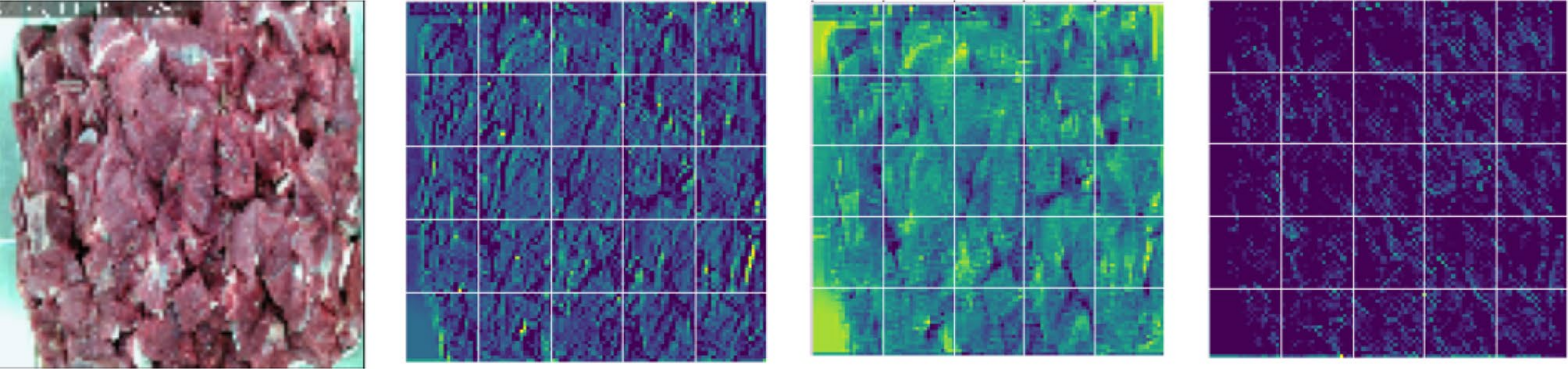
Some outputs of the first convolutional layer. Source: Authors' construction using Jupyter Notebook, and Python version 3.7.

The convolutional layers are the basis of CNN, which are employed as the feature extractor in this algorithm that these features discern images from each other. The neurons in these layers are arranged into feature maps. Neurons have a receptive field in a feature map connected to a neurons' neighborhood in the previous layer^38^. Figure 3 illustrates the outputs of the first convolutional layer in the proposed DCNN model by extracting the features of the image, leading to training an appropriate DCNN model.

Rectified linear unit (ReLU) is an activation function that applies non-linearity to the network, which this non-linearity helps produce the non-linearity boundaries. The introduction of this non-linearity to networks makes CNN an accurate algorithm. ReLU activation function is formulated in Eq. (3), and the output of a convolutional layer and ReLU is calculated using Eq. (4).

3 $$ReLU=Max (0, x)$$

4 $${Y}_{n}=f({W}_{n}*x)$$

where ${Y}_{n}$ is the *nth* output of feature map, *f* is activation function, which is *ReLU* here, *x* is the input image, ${W}_{n}$ is the convolutional filter related to the *nth* feature map, and the multiplication sign refers to the 2D convolutional operator.

Pooling layers have various types––decreasing the network's parameters and computations by reducing the network's spatial extent is the purpose of these layers types. The Batch-Normalization layer applies the batch normalization method, which converts the inputs to a mean of zero and a standard deviation of one^39^. The DCCN model training process is accelerated by this method. Dropout is a technique in which a percentage of randomly selected neurons are ignored. In other words, the contribution of these neurons is removed temporally on the forward propagation, and weight updating is not applied on the backward pass^40^. This technique helps avoid overfitting (i.e., the predictive model learns well but cannot predict correctly). The flatten layer transforms a three-dimensional input into a one-dimensional vector to prepare it for the fully connected layer. The fully connected is placed after the several convolutional and pooling layers to interpret and represent the features that have been extracted^41^. There are two classes in the binary classification problem; therefore, the Sigmoid activation function is utilized. The sigmoid function is formulated in Eq. (5) as:

5 $$sigmoid\left(X\right)= \frac{1}{1+ {e}^{- X}}$$

Several optimization algorithms were developed based on the stochastic gradient descent (SGD) algorithm, such as root means square propagation (RMSProp), adaptive moment estimation (Adam), and adaptive gradient algorithm (AdaGrad). In other words, these algorithms are extensions of the SGD algorithm. Adam is an optimization algorithm roughly combining AdaGrad and RMSProp algorithms^42^. Adam takes both algorithms superior, which uses the squared gradients to scale the learning rate like RMSProp and utilize the moving average of the gradient like AdaGrad. Adam updates the weights in a way formulated in Eq. (6).

$${m}_{t+1}\leftarrow {\beta }_{1}{m}_{t} + (1 - {\beta }_{1}) \nabla {C}_{t}$$

$${v}_{t+1}\leftarrow {\beta }_{2}{v}_{t} + (1 - {\beta }_{2}) {(\nabla {C}_{t})}^{2}$$

$$\widehat{m} = \frac{{m}_{t+1}}{1 - {\beta }_{1}^{t + 1}}$$

$$\widehat{v} = \frac{{v}_{t+1}}{1 - {\beta }_{2}^{t + 1}}$$

6 $${w}_{t+1} \leftarrow {w}_{t} - \eta \frac{\widehat{m}}{\sqrt{\widehat{v} } + \epsilon }$$

where $\epsilon$ is a small scalar, *m* is the first moment (i.e., m is mean), ${\beta }_{1}$ and ${\beta }_{2}$ are hyperparameters, *v* is the second moment (i.e., *v* is uncentered variance), *w* is model weight, $\eta$ is the learning rate (step size), and *C* is the cost function. Adam optimizer helps to strengthen the training efficiency and learning progress^43^.

### Architecture of the proposed DCNN model

**Figure 4:**
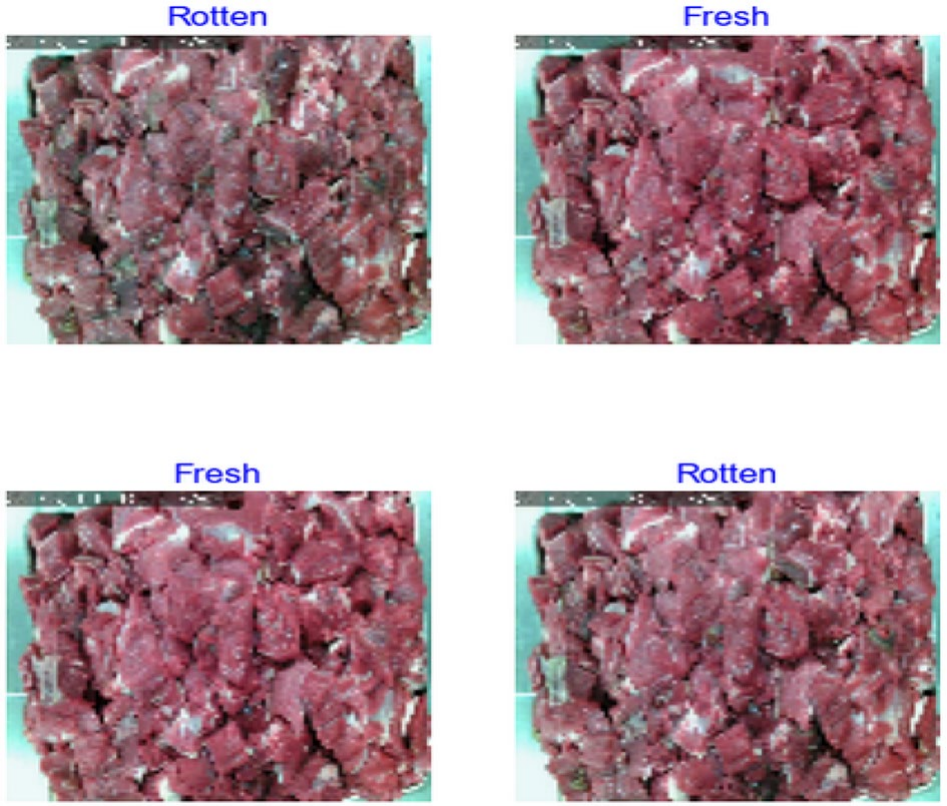
Samples of the dataset classes. Source: Authors' construction using Jupyter Notebook, and Python version 3.7.

The DCNN model includes feature extraction and classification. The process starts by resizing the image to 100 × 100 × 3, which 100 × 100 represents the height and width whereas × 3 refers to the number of color channels (i.e., Red, Green, and Blue). Figure 4 illustrates some samples of the dataset. The dataset contains two classes, which are fresh and rotten meat, which are extracted from existing dataset (i.e., Ulucan, Karakaya^36^).

The dataset is split into the training set (90%), validation set (5%), and test set (5%), so the training set contains 1706 images, whereas the test set and validation set included 95 photos each. Table 1 shows the number of samples assigned to each class used in training. Here, the amounts are unbalanced; therefore, each weight class is calculated and employed in the DCNN training process.

**Table 1:** Number of samples and their weights in the training set.

Class	Number of samples	Weight
Fresh meat	849	1.01
Rotten meat	857	1

The previous section showed the proposed layers that make the DCNN. Table 2 shows the proposed configuration of the model. The PSO optimization process selects the number of filters in convolutional layers and the learning rate.

**Table 2:** The configuration of the proposed model.

Layer	Receptive field size
Conv2D (ReLU)	3 × 3
BatchNormalization	–
Separable Conv2D (ReLU)	3 × 3
MaxPooling2D	2 × 2
BatchNormalization	–
Dropout (0.3)	–
Separable Conv2D (ReLU)	3 × 3
Separable Conv2D (ReLU)	3 × 3
BatchNormalization	–
MaxPooling2D	2 × 2
Dropout (0.4)	–
Conv2D (ReLU)	3 × 3
Conv2D (ReLU)	3 × 3
BatchNormalization	–
MaxPooling2D	2 × 2
Dropout (0.5)	–
Flatten	–
Dense (128 n*) (ReLU)	–
Dropout (0.3)	–
Dense (1 n) (Sigmoid)	–

*n number of neuron.

### Evaluation metrics

There are various criteria to evaluate the proposed model, such as Precision, Recall, F1-score, and Accuracy. The Precision, Recall, F1-score, and Accuracy metrics are formulated in Eqs. (7)–(10) as:

7 $$Precision = \frac{TP}{TP + FP}$$

8 $$Recall = \frac{TP}{TP + FN}$$

9 $$Accuracy = \frac{TP + TN}{TP + TN + FP + FN}$$

10 $$F1-score = \frac{TP}{TP + \frac{1}{2}( FP + FN)}$$

where True Positive (*TP*) represents the predictive model predicted positive, and the primary value is positive, True Negative (*TN*) indicates the predictive model predicted negative, and the primary value is negative, False Positive (*FP*) refers to the predictive model predicted positive, but the primary value is negative (Type 1 error), and False Negative (*FN*) demonstrates the predictive model predicted negative, but the primary value is positive (Type 2 error).

## Numerical results and discussion

In this section, the PSO algorithm adjusts two critical hyperparameters, namely learning rate and number of filters in the convolutional layer. The DCNN model is trained, and several evaluation metrics are utilized to assess the model performance.

### Parameters tuning by PSO algorithm

Learning rate is a significant hyperparameter in deep learning models that controls the model changes in response to the estimation error of updating model weights^44^. If the amount of the learning rate selected is too small, the training process gets extended or even get stuck. In contrast, a too large amount will lead to an unstable training process and unproperly weight updating^44^. The number of different ways of extracting features from an image is determined based on the number of filters in the convolutional layers^45^. The more filters, the more features can be extracted, but this rule is not always proper for CNN models; therefore, the number of filters must be adjusted. Thus, the PSO algorithm, a popular population-based optimization method, is utilized to adjust the number of filters in convolutional layers and the learning rate in the proposed DCNN model. The detailed configuration of the proposed DCNN model is denoted in Table 3, in which the PSO algorithm achieved the number of filters for the convolutional layers, and the learning rate was earned 0.001 among 1, 0.1, 0.01, and 0.001 by the PSO too.

**Table 3:** The detailed configuration of the proposed DCNN model.

Layer	NF*	Padding*	RFS*	AF*	NN*	DR*
Conv2D	32	Same	3 × 3	ReLU		
BatchNormalization						
Separable Conv2D	32	Same	3 × 3	ReLU		
MaxPooling2D			2 × 2			
BatchNormalization						
Dropout						0.3
Separable Conv2D	64	Same	3 × 3	ReLU		
Separable Conv2D	64	Same	3 × 3	ReLU		
BatchNormalization						
MaxPooling2D			2 × 2			
Dropout						0.4
Conv2D	128	Same	3 × 3	ReLU		
Conv2D	128	Same	3 × 3	ReLU		
BatchNormalization						
MaxPooling2D			2 × 2			
Dropout						0.5
Flatten						
Dense					128	
Dropout						0.3
Dense				Sigmoid	1	

*NF number of filters, *RFS* receptive field size, *AF* activation function, *NN* number of neurons, *DR* dropout rate.

### Evaluation of the proposed DCNN model

**Figure 5:**
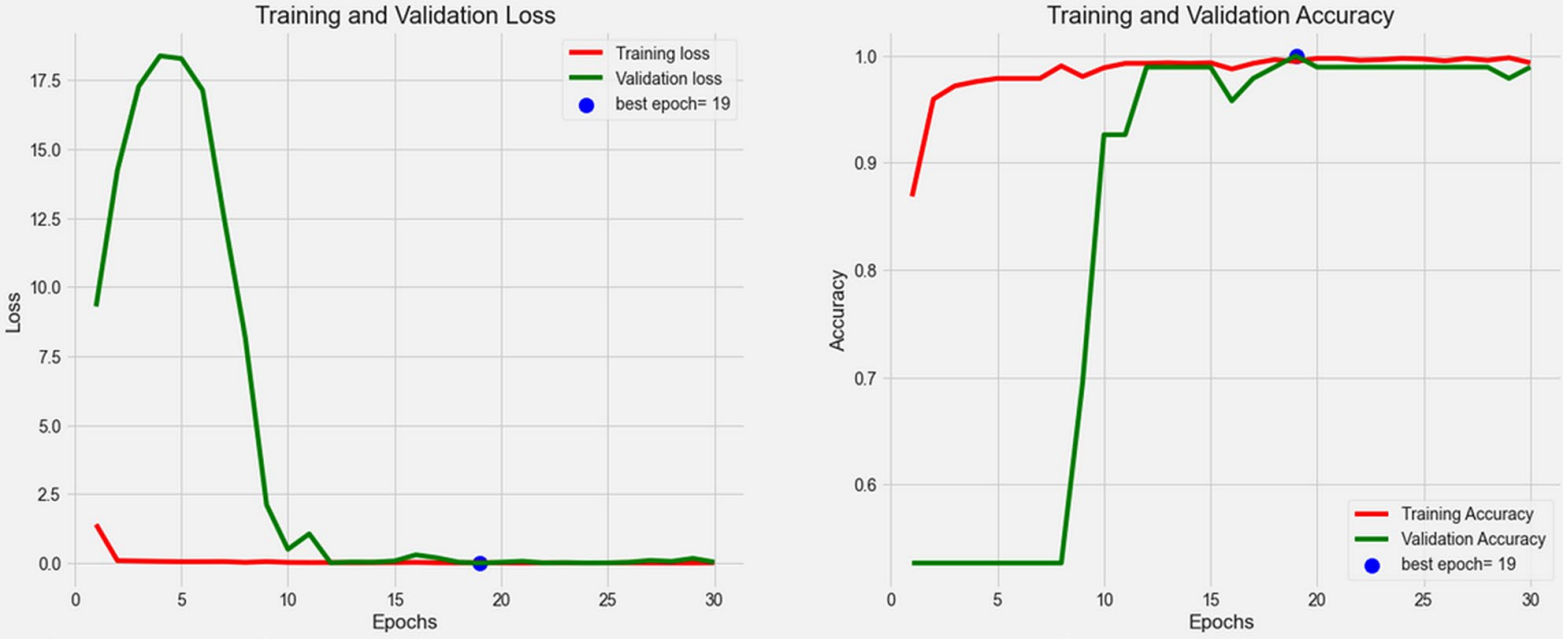
The training and validation process of the DCNN model. Source: Authors' construction using Jupyter Notebook, and Python version 3.7.

The classifier model is built based on the configuration mentioned in Table 3, the number of filters and the learning rate that the PSO achieved. Figure 5 shows the training and validation process of the DCNN model.

**Figure 6:**
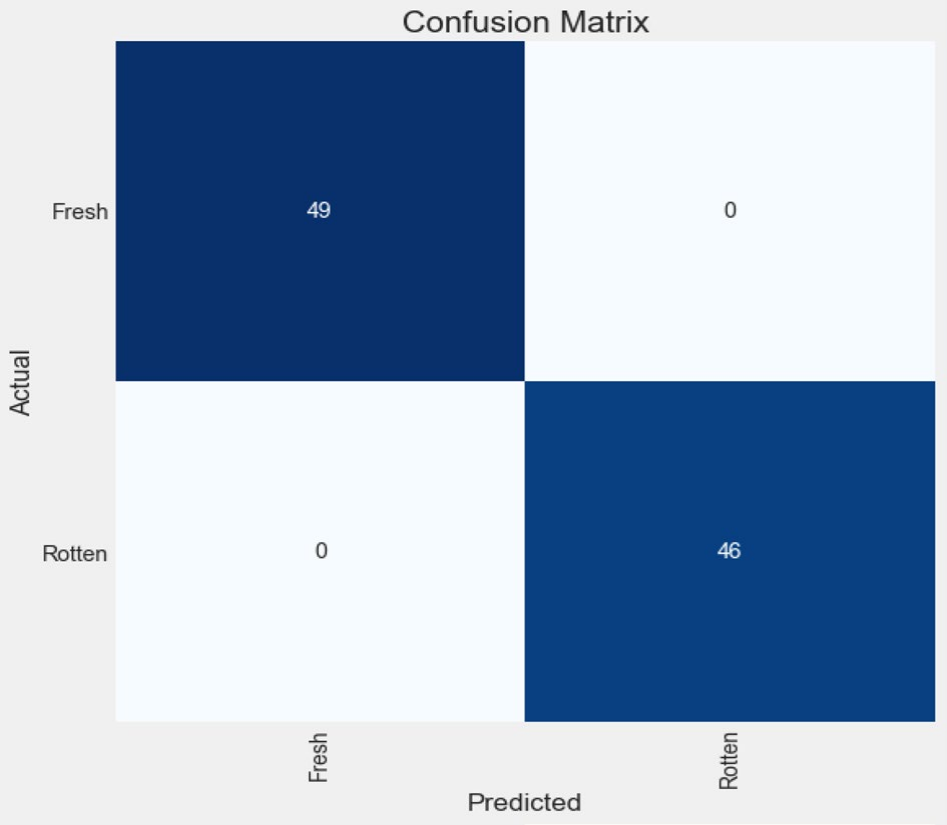
The confusion matrix result. Source: Authors' construction using Jupyter Notebook, and Python version 3.7.

Some evaluation metrics such as the confusion matrix, Precision, Recall, F1-score, and Accuracy are employed to assess the predictive model. Figure 6 illustrates the results of the confusion matrix, whereas other metrics are presented in Table 4.

**Table 4:** The model evaluation result.

Class	Precision (%)	Recall (%)	F1-score (%)	Accuracy (%)
Fresh meat	100	100	100	100
Rotten meat	100	100	100	100

A comparison between the output of this study and existing literature, viz. Ulucan, Karakaya^36^ is outlined in Table 5, which shows the results achieved in this paper have better performance than previous literature.

**Table 5:** Comparison of this paper with the existing literature.

Research	Accuracy (%)
This paper	100
Ulucan, Karakaya^36^	99.62

## Discussion

Our model proposed an intelligence method that is more efficient in reducing human error and losses while increasing the accuracy and availability in various sections of the meat supply chain than traditional monitoring and control systems that are considered in previous studies^46–49^. Moreover, the model has better performance than the previous research^36^ on deep learning model by utilizing PSO algorithm and proper model architecture. This research provided a DCNN and PSO algorithm to train an image classifier system to control and distinguish wholesome meats from spoiled ones. The PSO algorithm was utilized to tune two critical features (i.e., learning rate and the number of filters in convolutional layers) that significantly affect the performance of DCNN models. One of the common problems in image classification problems is overfitting (i.e., model is trained well but cannot predict appropriately); therefore, by using the correct architecture and Dropout method, the DCNN model, as shown in Fig. 5, appears more robust without this problem. Several metrics, namely accuracy, Precision, Recall, and F1-score––which are calculated based on the Confusion matrix, were applied to assess the DCNN model performance and indicate the model efficiency. Based on the results outlined in Table 4, the DCNN model with 100% accuracy has remarkable performance.

The industry 4.0 and SDG12 paradigms recommend utilizing new technologies such as artificial intelligence in manufacturing and supply chain management to increase productivity and decrease expenses and losses. In this paper, the deep learning model was employed to automate the controlling and separation process of wholesome meats from spoiled ones. This system can be utilized in transportation, storage, and retail sections in the meat supply chain to control and monitor the meats. This process is necessary to reduce bacteria effects in spoiled meat that may contaminate wholesome meats, hence, improving shelf life and saving final customers from harmful risks due to food spoilage. This system displaces manual monitoring and controlling; hence, it can be active most often and mitigate human errors, leading to enhanced shelf life while decreasing losses and increasing productivity, which are the goals of industry 4.0 and SDG12 paradigms.

## Conclusion

This paper proposed the application of artificial intelligence in meat supply chain management based on industry 4.0 and SDG12 paradigms. A classifier was trained by the DCNN and PSO algorithms with 100% accuracy, which distinguishes wholesome meats from spoiled ones. This model is utilized in various steps of the meat supply chain, which increases productivity, reduces cost, and avoids the bacteria effects of rotten meats on healthy ones by automating the separation process. Besides, by enhancing the meat shelf life, consumer confidence and preferences for meat can increase, hence, increasing economic productivity. For future research, this system can be employed for more perishable products, as well as including other technologies (i.e., IoT) to the proposed system to control other factors, including humidity and temperature. Moreover, artificial intelligence techniques can be considered for different tasks of supply chain management, for instance, product packaging and demand forecasting.

## Data Availability

Data sets analyzed during the current study are available from the current author on request.
